# Whole Sets of Teeth.—No. III

**Published:** 1850-10

**Authors:** W. E. Ide

**Affiliations:** Columbus


### WHOLE SETS OF TEETH.—NO. III.

BY W. E. IDE, OF COLUMBUS.

In No. II* of this series I gave in detail my method of taking impressions for whole sets of teeth. As nearly two years have elapsed since that number was written, it may not seem strange that in many particulars my practice has changed. In no case with my present experience would I take an impression for an entire *upper* set of teeth in any other material than Plaster of Paris. Indeed, it is the only material that can by any possibility give a perfect representation of the soft, as well as the hard parts of the jaw. If skillfully done, each corrugation

*Nos. I and 2 of this series will be found in first volume Dental Register.—Ed.

and reflection of mucous membrane *in situ*, is made to yield its unerring *fac simile.*

My method is as follows: A plate of tin *a little larger* than the jaw, is selected from an assortment that has been *struck up* from dies used in previous cases. A small quantity of freshly calcined plaster is mixed with pure rain water and a little table salt, to the consistence of batter. A part of this is spread with a spatula on the palatine arch, and the balance poured into the tin plate, and immediately introduced into the mouth and pressed upon the jaw.

For a minute description of this process, I would refer the reader to a well written article on the subject, by Dr. Dwinelle, in the Dental Journal of October, 1848, and republished in the Register of April, 1849, with some judicious remarks by Prof. Taylor. I would only add that if the plaster is a good article, and freshly calcined, considerable dexterity is required to get through with the manipulation before the hardening process commences; and, also, that the saliva should be thoroughly removed from the jaw by a napkin before the plaster is applied; as its presence interferes with the hardening process, and makes a rough surface.

The impossibility of avoiding the presence of saliva, and the necessity of displacing the membranous reflections have induced me in all cases to prefer wax for taking impressions of the *lower* jaw. In this case I proceed in the usual way, by pouring plaster into the wax, separating, trimming, building out with wax to relieve the muscular attachments, varnishing, moulding in sand and casting the dies in zinc. In this I claim no peculiarity, but with the upper jaw my mode of procedure differs essentially from that in common use.

Having removed the plaster impression from the mouth, I proceed to carve in it a crescent-shaped depression about a line deep, and the size of half an American twenty-five cent piece. This should be done on the part corresponding with a point on the posterior side of the alveolus and extending to one on the center of the palatine arch. This, it will be perceived, is for the purpose of making an air-chamber.

The plaster thus prepared is placed upon a small piece of shingle or board, and around it is set a cast iron ring of a diameter somewhat greater than the plaster impression, about three inches high and having a smooth and even inner surface. The space between the circumference of the plaster and the ring is filled with moistened sand, and the whole placed carefully in a stove to dry. When this is accomplished the whole is filled with melted type metal, which, after cooling, is a perfect stereotype representation of the parts to be covered by a suction plate. This being inverted and imbedded in sand up to a point nearly as high as that which represents the alveolar arch, is again surrounded by the cast iron ring and type metal of the same quality, but of a temperature as low as is consistent with perfect fusion, is poured upon it till the ring is again full. Some care and dexterity in pouring the metal is neceessary to prevent adhesion; but it is readily accomplished by having the first cast quite cold, the melted metal of a low temperature as above directed, and avoiding a continuous stream on any one particular part. I use type metal because of the low degree of heat required for fusion, and because there is less contraction in cooling than in any other metal. A small quantity of lead may be added in case it may be necessary to have something less brittle, as when from the shape of the male cast there may be a difficulty in the separation. The cast having been thus obtained, we next proceed to make a copy as exact as possible by moulding it in sand, and casting in zinc, precisely as is usually done from a plaster model. Upon the zinc, lead is poured, which being done, we have two sets of dies, the one absolutely perfect, and the other as near an approximation as can be made in the usual mode.

The plate is first struck up on the zinc and lead dies, by repeatedly annealing and striking till it is made to fit them perfectly, and is then finished upon the type metal dies. It not unfrequently happens that the jaw recedes above the front alveolar border in such a manner as to render it impossible to draw the cast from the sand, without adding wax, and thereby spoiling that part of the fit. The wax can be added to the first cast so that it can be drawn from the sand and the fit will be perfected, at the time of the last striking, so that no application of the pliers need ever be necessary.

In my next will be given my method of arranging the teeth, articulating and finishing.

